# The Genome Sequence of a Widespread Apex Predator, the Golden Eagle (*Aquila chrysaetos*)

**DOI:** 10.1371/journal.pone.0095599

**Published:** 2014-04-23

**Authors:** Jacqueline M. Doyle, Todd E. Katzner, Peter H. Bloom, Yanzhu Ji, Bhagya K. Wijayawardena, J. Andrew DeWoody

**Affiliations:** 1 Department of Forestry and Natural Resources, Purdue University, West Lafayette, Indiana, United States of America; 2 Department of Biological Sciences, Purdue University, West Lafayette, Indiana, United States of America; 3 Division of Forestry and Natural Resources, West Virginia University, Morgantown, West Virginia, United States of America; 4 Northern Research Station, USDA Forest Service, Parsons, West Virginia, United States of America; 5 Western Foundation of Vertebrate Zoology, Camarillo, California, United States of America; Natural History Museum of Denmark, University of Copenhagen, Denmark

## Abstract

Biologists routinely use molecular markers to identify conservation units, to quantify genetic connectivity, to estimate population sizes, and to identify targets of selection. Many imperiled eagle populations require such efforts and would benefit from enhanced genomic resources. We sequenced, assembled, and annotated the first eagle genome using DNA from a male golden eagle (*Aquila chrysaetos*) captured in western North America. We constructed genomic libraries that were sequenced using Illumina technology and assembled the high-quality data to a depth of ∼40x coverage. The genome assembly includes 2,552 scaffolds >10 Kb and 415 scaffolds >1.2 Mb. We annotated 16,571 genes that are involved in myriad biological processes, including such disparate traits as beak formation and color vision. We also identified repetitive regions spanning 92 Mb (∼6% of the assembly), including LINES, SINES, LTR-RTs and DNA transposons. The mitochondrial genome encompasses 17,332 bp and is ∼91% identical to the Mountain Hawk-Eagle (*Nisaetus nipalensis*). Finally, the data reveal that several anonymous microsatellites commonly used for population studies are embedded within protein-coding genes and thus may not have evolved in a neutral fashion. Because the genome sequence includes ∼800,000 novel polymorphisms, markers can now be chosen based on their proximity to functional genes involved in migration, carnivory, and other biological processes.

## Introduction

For millennia, eagles have been cultural icons emblematic of nations, religions, and peoples around the world ([1], [2]; Figure S1). In ancient Egypt, eagle hieroglyphs were symbolic of the soul after death. In contemporary North America, native cultures incorporate eagle feathers into medicines and religious ceremonies. Eagles have long been trained for falconry in Central Asia and are still used to hunt prey as large as wolves in Mongolia [2].

Eagles are also apex predators whose trophic impacts cascade through ecosystems, as their prey range in size from beetles to marine mammals and span a gamut that includes frugivores, herbivores, carnivores, omnivores, and planktivores (e.g., monkeys, deer, hawks, tortoises, fishes, etc.) [3]–[7]. Unfortunately, many eagle species are of worldwide conservation concern due to direct threats to individuals (e.g., poaching and collisions with wind turbines) and indirect threats to populations (e.g., habitat loss and environmental toxins) [2], [8]–[11]. Conservation efforts have often been hampered by the generally secretive nature and remote habitats of eagles, but recently described molecular markers have provided new tools for population monitoring [12], [13]. Modest suites of microsatellite markers are now available for a few species (e.g., *Aquila adalberti*, [14]); *A. heliaca*, [15]; *Haliaeetus albicilla*, [16]; *Nisaetus nipalensis*, [17]), and complete mitochondrial genome sequences are available for three species (*Spilornis cheela* [18], *N. nipalensis,* and *Spizaetus alboniger* [19]).

Avian genomics, however, still lags far behind mammalian genomics as scores of complete mammalian genomes have been sequenced, but only about a dozen avian genomes have been published (Table 1). With this in mind, we sequenced the genome of the golden eagle (*Aquila chrysaetos*) to facilitate comparative studies of avian genomics and to further the development of genetic tools for eagle research and conservation. Golden eagles are among the most widespread of avian species, with a distribution that spans the Paleartic and Nearctic and extends into the Afrotropic and Indomalaya ecozones [2]. They are often considered a mountain resident, but can thrive in an array of habitats including shrub-steppe communities, deserts, bogs, peatlands and tundra [2]. Nevertheless, the golden eagle is threatened throughout much of its range. Historical and ongoing population declines and a suite of persistent and novel threats have led to governmental protection of these birds in much of their range [2], [10], [20]–[22].

**Table 1:** Assembled avian nuclear genomes in NCBI as of 12 September 2013.

Species	Order	Assemblysize (Mb)	Estimatedgenomesize^a^	Estimated#genes	Meangenelength	Meanexonspergene	Meanexonlength	Meanintronlength
***Aquila chrysaetos*** ** (Golden Eagle)**	**Falconiformes**	**1548.5**	**1.28, ** ***1.32*** **, ** ***1.48*** [59], [60]	**16,571**	**25049**	**8.6**	**143**	**2646**
*Amazona vittata* (Puerto Rican Parrot) [82]	Psittaciformes	1175.4	*1.58* [83]	-	-	-	-	-
*Anas platyrhynchos* (Mallard) [57]	Anseriformes	1105.1	1.26	19,144	20574	8.2	164	2664
*Ara macaeo* (Scarlet Macaw) [71]	Psittaciformes	1204.7	*1.34* [59]	14,405	-	-	-	-
*Falco peregrinus* (Peregrine Falcon) [36]	Falconiformes	1172.0	1.22	16,263	20646	8.9	173	2395
*Falco cherrug* (Saker Falcon) [36]	Falconiformes	1174.8	1.19	16,204	19314	8.8	173	2250
*Ficedula albicollis* (Collared Flycatcher) [84]	Passeriformes	1118.3	1.31	18,649	-	-	-	-
*Gallus gallus* (Chicken) [64]	Galliformes	1046.9	*1.24* [59]	17,040	16702	8	166	2203
*Geospiza fortis* (Medium Ground-finch) [85]	Passeriformes	1065.3	1.25	13291	-	-	-	-
*Meleagris gallopavo* (Turkey) [86]	Galliformes	1061.8	*1.31* [87]	15,704	-	-	-	-
*Melopsittacus undulatus* (Budgerigar)	Psittaciformes	1117.4	*1.22* [88]	-	-	-	-	-
*Pseudopodoces humilis* (Tibetan Ground-tit) [89]	Passeriformes	1043.0	1.08	17,520	19840	9.27	170	2208
*Taeniopygia guttata* (Zebra Finch) [72]	Passeriformes	1232.1	*1.25* [90]	14527	-	-	-	-
*Columbia livia* (Rock Pigeon) [50]	Columbiformes	1108.0	1.3	17,300	18364	8.5	166	2271
*Mean assembly size*		1143.0						

a Genome sizes in plain text were estimated by the kmer method (citations can be found in the “species” column). Genome sizes in italics were estimated by other methods (e.g., Feulgen absorption microspectrophotometry; citations can be found in the “estimated genome size” column).

A complete sequence of the golden eagle genome can facilitate the conservation of this species in a number of ways. For example, a major source of mortality to golden eagles is collision with wind turbines and other structures [2], [10]. Scientists have hypothesized that raptors might be better able to avoid these structures if they were coated with ultraviolet-reflective paint [23]. The color vision system is undescribed in golden eagles, however. The golden eagle genome sequence can be used to determine whether the color vision system is violet-tuned or ultraviolet-tuned, shedding light on whether UV-reflective paint has potential merit. Furthermore, a complete sequence of the golden eagle genome will prove valuable for those interested in the evolution, ecology, and demography of this charismatic species by virtue of the molecular polymorphisms contained therein.

## Methods

Here, we provide a broad overview of our methods. Further details are available in the Electronic Supplementary Materials (ESM) available online at the journal’s website.

### Sampling, Molecular Methods, and Quality Control

**Figure 1:**
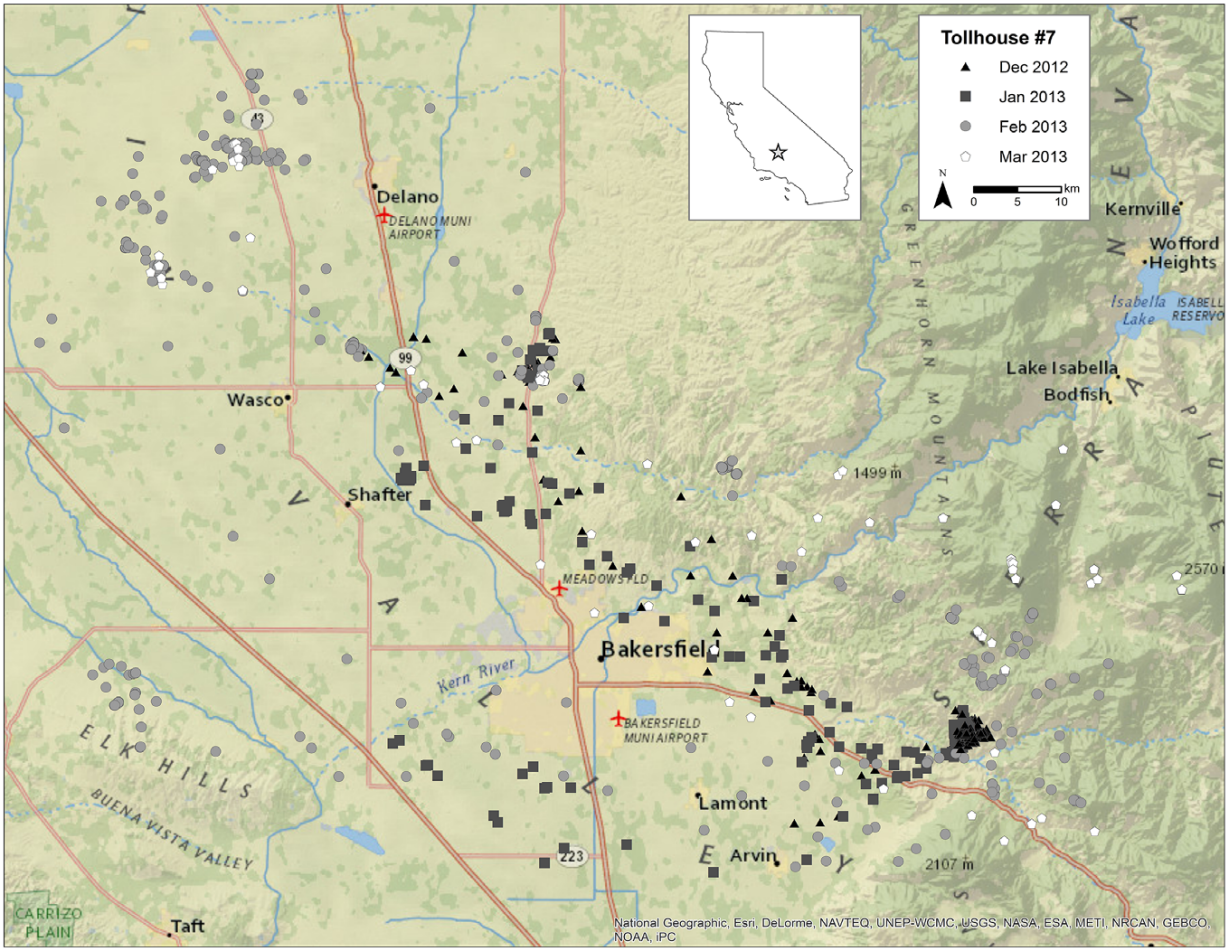
Movements of the captured male golden eagle. Movements of the golden eagle (USFWS Band #0679-02608) whose genome sequence is presented herein. GPS data were collected by a CTT-11060 telemetry unit at 15-minute intervals from capture date (6 December 2012) through 07 March 2013. Home range size during this period was 1068 km^2^ (95% KDE).

A male golden eagle (subspecies *A. c. canadensis*) was captured 6 December 2012 in the California foothills of the southern Sierra Nevada, between the Central Valley and the Mojave Desert (N 35 18 29.2 W 118 38 05.7). The propositus was captured with a bow net following approved protocols (West Virginia University’s Animal Care and Use Committee, protocol #11-0304) and under federal and state bird banding permits (BBL#20431; Cal SCP #SC-221) [24]. Three drops of blood (∼2 ml) were collected via venipuncture of the brachial vein were preserved in 1 ml of lysis buffer (100 mM Tris-HCl, 100 mM EDTA, 10 mM NaCl, 2% SDS) and the eagle was outfitted with a GPS-GSM tracking device [24] before release (Figure 1). Genomic DNA was subsequently extracted using a standard phenol chloroform protocol [25] and a standard PCR assay was used to confirm sex genetically [26].

In February and March 2013, we conducted one lane of paired-end sequencing and one lane of mate-paired sequencing using an Illumina HiSeq2000 that produced read lengths of 100 bp. Quality control included a) adaptor removal using Trimmomatic ([27], Table S1 in File S1); b) discarding short reads (<30 bp); c) trimming poor quality bases (Illumina Q-value≤20) from both 5′ and 3′ ends of raw sequence reads; and d) removing all identical paired-end reads (i.e., PCR duplicates).

### Genome Assembly and Genome Size Estimation

We used ABySS [28] for *de novo* assembly of the *A. chrysaetos* nuclear genome. We used trimmed paired-end reads and mate-paired reads (as single-end reads) to create consensus sequences. Briefly, all possible K-mers were generated from sequence reads and a de Bruijn graph [28] was created by joining overlapping K-mers. Subsequently, both paired-end and mate-paired data were used to resolve ambiguities among contigs and to link contigs into scaffolds. The completeness of the assembly was assessed by CEGMA, which assesses the proportion of proteins predicted from the *A. chrysaetos* genome relative to a conserved set of core eukaryotic proteins [29].

We used the K-mer approach to estimate total genome size. Briefly, we used Jellyfish [30] to divide all paired-end sequenced reads into K-mers of 17 nucleotides and to plot the frequency of each K-mer so that the peak depth represented the mean K-mer coverage (M) of the genome (Figure S2). We then estimated the actual coverage of the genome (N) using the equation N = M/((L−K+1)/L), where L is the mean read length and K is the K-mer size [31]. Sequence coverage was estimated by dividing total sequence data by genome size.

For assembly of the *A. chrysaetos* mitochondrial DNA (mtDNA) genome, we first used the Mountain Hawk-Eagle (*Nisaetus nipalensis*; Asai et al., 2006) mtDNA genome as a reference to map our paired-end reads using Bowtie2 [32]. We also used MITObim, which employs a baiting and iterative mapping approach [33].

### Gene Annotation

The *A. chrysaetos* mtDNA genome was annotated using DOGMA [34] and visualized with OGDraw [35]. To help annotate the *A. chrysaetos* nuclear genome, we used EST and protein evidence from other avian species. We downloaded *Gallus gallus*, *Meleagris gallopavo, Taeniopygia guttata* and *Columba livia* protein sequences from the UniProtKB database (www.uniprot.org) and *Falco cherrug* RNAseq reads from the NCBI short read archive [36]. The RNA-seq reads were assembled *de novo* into contigs using Trinity [37] after employing the quality control measures described earlier. We then used the pipeline MAKER [38], which incorporates the following programs (among others): 1) RepeatMasker [39] which identified and masked stretches of repetitive DNA in the eagle genome; 2) BLAST, which aligned avian ESTs and proteins to the genome; and 3) SNAP [40] and AUGUSTUS [41], which produced *ab initio* gene predictions for *A. chrysaetos*. MAKER synthesized these data and produced final annotations with evidence-based quality values. MAKER was run in an iterative manner such that gene models from one run acted as inputs for subsequent runs. The initial evidence used in MAKER included the 415 *A. chrysaetos* genome sequences greater than 1.2 Mb in length (Table S2 in File S1) and the 2,385 protein sequences from *Gallus gallus*, *Meleagris gallopavo, Taeniopygia guttata* and *Columba livia*. The protein2genome setting in MAKER was used to produce gene annotations directly from protein evidence, and this output file was used to train SNAP. We then completed a second MAKER run using the same initial evidence, but the protein2genome setting was not used. The results were then used to train SNAP a second time. In the third iteration, we supplied MAKER with 1) 2,552 *A. chrysaetos* genome sequences greater than 10.0 Kb; 2) all 2,385 avian protein sequences; and 3) 234,818 ESTs (i.e., RNAseq contigs) from *Falco cherrug*. We ran AUGUSTUS with the “chicken” species setting and RepeatMasker with the “all” setting.

Given our heterospecific libraries of protein and EST evidence, we initiated a second pipeline to identify genes that remained unannotated. We collected all SNAP and AUGUSTUS *ab initio* gene predictions that were not supported by EST or protein evidence and used InterProScan to identify putative protein domains. Accordingly, gene predictions containing presumptive protein domains were promoted to gene annotations, and InterProScan was used to assign ontologies to each gene. In order to compare our results to those of other studies, we also used InterProScan to assign ontologies to saker and peregrine falcon genes [42].

### Xenobiotics and Repetitive Sequences

All of our sequences were derived from genomic libraries constructed from bird blood, but this does not mean that all sequences are of eagle origin. We delineated xenobiotic sequences to identify potential pathogens, parasites, and commensals of *A. chrysaetos.* First, all contigs longer than 200 bases were used as BLAST queries (BLASTN parameters; E value = 1E-6) against the chicken genome (ensembl database: Gallus_gallus.Galgal4.72.dna.toplevel.fa) to identify known avian sequences. Subsequently, all remaining contigs (i.e., those very dissimilar to chicken) were extracted and used as BLAST queries (BLASTN parameters; E value = 1E-6) of the entire GenBank catalog. For each of these query sequences, up to 1000 hits were collected and the sequence was categorized as either vertebrate or invertebrate in origin. Contigs that matched no vertebrate taxa were identified as putative xenobiotics (Table S3 in File S1).

After excluding the xenobiotic contigs, repetitive elements in the *A. chrysaetos* assembly were detected by a combination of methods, including homology-based and *de novo* approaches [43]–[46]. We used RepeatMasker [39], RepeatProteinMask [39] and RepeatModeler [47] to identify interspersed repeats, then ran Tandem Repeats Finder [48]. Custom perl scripts (modified from L. Hu, personal communication) were used to remove overlapping regions and calculate overall repeat content.

### Linkage Disequilibrium and Molecular Markers

The extent of linkage disequilibrium (LD) in avian species is known to vary between 0.5–400 Kb ([49] [50]). Bourke and Dawson [51] described fifteen anonymous microsatellites from the *A. chrysaetos* nuclear genome. We used a custom perl script to identify their primer sequences in our scaffolds, then used the program Apollo [52] to locate genes within 400 Kb in an effort to determine which of these 15 markers might be most heavily influenced by hitchhiking associated with selective sweeps.

To extend the suite of *A. chrysaetos* molecular markers, we used the genome assembly to identify additional microsatellites using MISA [53]. Single nucleotide polymorphisms (SNPs) were identified using Bowtie2 [33] to align all filtered paired-end reads to contigs longer than 200 bases. Samtools [54] was subsequently used to call SNPs with coverage greater than 10 reads and less than 60 reads, with a quality score of 20 or better, in order to compare our results to that of other studies (e.g., peregrine and saker genomes [36]).

### Color Vision Determination

Avian color vision can be categorized as violet or ultraviolet, and associated sensitivity can be determined from the SWS1 opsin protein sequence [53]. We downloaded opsin sequences for three raptor species from NCBI (*Accipiter gentilis* AY227148; *Buteo buteo* AY227150; *Pandion haliaetus* AY227152 [55]). We used blastn to identify a single scaffold in our assembly that contained the SWS1 opsin coding region and used ExPASy to translate the nucleotide sequence to amino acid sequence.

## Results

We generated 68.4 Gb of raw sequence data from *A. chrysaetos*, including 25.3 Gb from the paired-end library and 43.1 Gb from the mate-paired library (Table S4 in File S1). Quality control filtering yielded 24.5 Gb and 21.0 Gb from the paired-end and mate-paired libraries, respectively, so about one-third of the raw data fell to the cutting-room floor [56]. More reads were filtered from the mate-paired data than the paired-end data because the cluster density associated with mate-paired data was higher. As cluster density increases, so too does interference from nearby clusters and therefore more reads are discarded by the clipping/filtering program.

**Figure 2:**
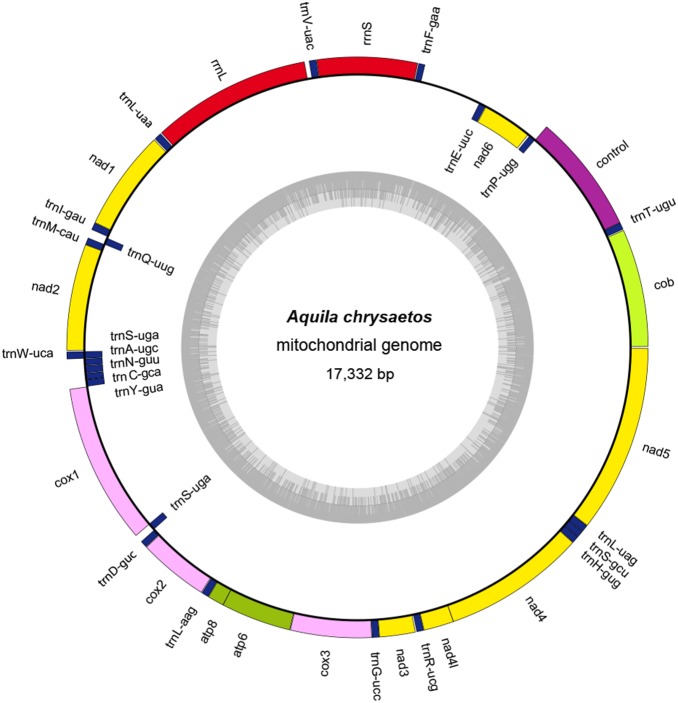
*A. chrysaetos* mitochondrial genome map. Cox1, cox2 and cox3 indicate cytochrome oxidase subunits 1–3; cob indicates cytochrome b; atp6 and atp8 indicate ATPase subunits 6 and 8; nad1–nad6 indicate NADH dehydrogenase subunits 1–6. Transfer RNA genes are designated by single-letter amino acid codes.

The MITObim assembly of the *A. chrysaetos* mtDNA genome produced a sequence of 17,332 bp (Figure 2), whereas the Bowtie2-produced genome was 17,647 bp. These assemblies were 97% identical to each other and, on average, were 92% identical to the *N. nipalensis* mtDNA genome. Given the strong concordance between the two approaches, hereafter we refer only to the MITObim assembly. The mtDNA genome is characterized by 13 protein-coding genes, two ribosomal subunit genes (rRNA), 23 transfer RNA genes (tRNA; Table S5 and Table S10 in File S1). Twenty-eight genes reside on the α-strand and 10 on the β-strand, and the putative control region is 1157 bp. As in most vertebrates, all protein-coding genes except NAD6 were found on the α-strand (Figure 2, Tables S5 and S10 in File S1).

**Figure 3:**
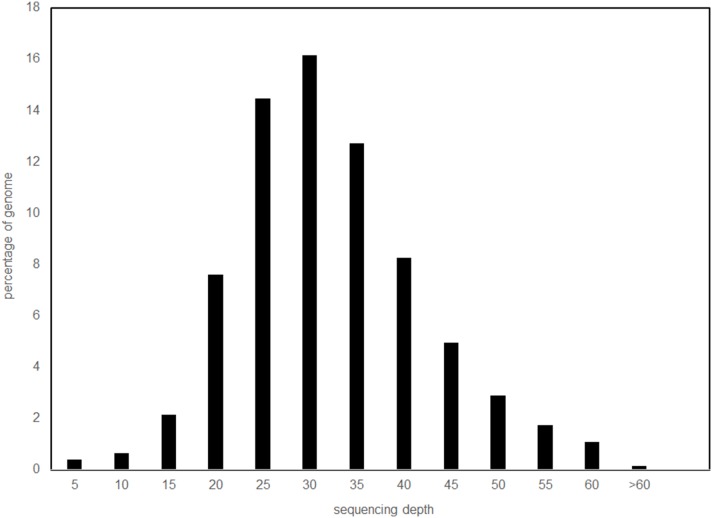
Depth of sequencing of the *A. chrysaetos* genome. Sequencing depth is on the x-axis while the y-axis shows the percentage of total bases at a given depth. Reads were aligned to the genome using bowtie2.

We divided our total paired-end sequence data (24,385,716,189 bp) by N to estimate a genome size of 1.28 Gb (including the mtDNA genome) and overall genome coverage was estimated as 38.9X (Figure 3, Table S4 in File S1). Nuclear genome assembly with ABySS produced 42,926 scaffolds that contain 1,548 Mb. These scaffolds had an N50 of 1,746,960 bp and the longest scaffold was 11,517,212 bp in length (Table S2 in File S1). Table S6 in File S1 indicates that approximately 90% of the core eukaryotic genes were identified in the *A. chrysaetos* genome.

EST and protein evidence greatly facilitates genome annotation. The 2,385 *Gallus gallus*, *Meleagris gallopavo, Taeniopygia guttata* and *Columba livia* protein sequences we used corresponded to 1,125,485 bases in total and had a N50 of 603. Our *de novo* assembly of the *Falco cherrug* transcriptome from RNA-seq reads produced 234,818 contigs that spanned 162,920,697 nucleotides, and contig length ranged from 101–17,136 bp with a N50 of 2,306.

Our comprehensive annotation of the *A. chrysaetos* genome produced a total of 16,571 predicted nuclear genes. Mean gene length was 25,049 nucleotides and on average, 8.6 exons were predicted in each gene. Mean exon and intron lengths were 143 bp and 2,646 bp, respectively. Based on protein domains, 89% of the *A. chrysaetos* genes were assigned gene ontologies and the top 100 protein domains can be found in Table S7 in File S1. We assigned gene ontologies to 79% and 80% of the saker and peregrine falcon predicted genes, respectively.

The total repeat content of the *A. chrysaetos* genome was estimated to be 5.94% (Table 2). Golden eagle repetitive elements are primarily composed of long interspersed nuclear elements (LINEs), then long terminal repeat retrotransposons (LTR-RTs), followed by DNA transposons and short interspersed nuclear elements (SINEs, Table 2). The total repeat content of the *A. chrysaetos* genome is most similar to the 5.86% found in mallard ducks ([57], Table S8 in File S1). Putative xenobiotic organisms represented in our sequence data are listed in Table S3 in File S1.

**Table 2:** Repetitive elements in the *A. chrysaetos* genome. Numbers indicate repeat size in bp and percentage of genome assembly (in parenthesis).

	Total	RepeatProteinMask	RepeatMasker	RepeatModeler	trf
	repeat size: bp (%)	repeat size: bp (%)	repeat size: bp (%)	repeat size: bp (%)	repeat size: bp (%)
SINEs	2,063,865 (0.13%)	NA	1,664,482 (0.11%)	773,136 (0.05%)	NA
LINEs	39,834,388 (2.57%)	22,041,715 (1.42%)	35,622,475 (2.30%)	28,613,532 (1.85%)	NA
LTRs	21,717,448 (1.40%)	2,619,141 (0.17%)	19,036,431 (1.23%)	17,744,753 (1.15%)	NA
DNAs	8,382,378 (0.54%)	256,301 (0.02%)	7,635,911 (0.49%)	1,412,454 (0.09%)	NA
Unknown	7,837,457 (0.51%)	0 (0.00%)	844,327 (0.05%)	6,993,212 (0.45%)	NA
Tandem repeats	14,577,786 (0.94%)	NA	588,197 (0.04%)	244,508 (0.02%)	14,109,713 (0.91%)
Total	92,021,614 (5.94%)	24,908,961 (1.61%)	64,751,314 (4.18%)	56,079,698 (3.62%)	14,109,713 (0.91%)

trf, Tandem Repeat Finder [46].

Each of Bourke and Dawson’s 15 microsatellites [51] were located in a genomic scaffold (Table S9 in File S1). Twelve were found within 400 kb of a gene, three within 20 kb of a gene, and two microsatellites were located in the noncoding regions of annotated genes (Table 3). Gene ontology terms associated with these genes ranged from metabolic process to tumor necrosis factor (Table 3).

**Table 3:** Proximity of anonymous microsatellites [51] to annotated *A. chrysaetos* genes.

Locus	Repeat motif	Scaffold length(nt)	Number of geneswithin			Notes	Ontologies of genes within 20 kb
			1 kb	10 kb	20 kb		
Aa43	(AC)_14_	385553	0	1	1		Exostosin
Aa15	(CA)_13_	201766	1	2	3	Microsatellite within gene	Metabolic process
Aa26	(AC)_14_	498563	0	0	0		
Hal10	(CA)_12_	897486	0	0	0	Nearest gene within 430 kb	
IEAAAG09	(RAAG)_18_	750407	0	0	0	Nearest gene within 306 kb	
Aa11	(CA)_11_	211691	0	0	0	Nearest gene within 63 kb	
Aa36	(AC)_16_	173020	0	0	0	Nearest gene within 215 kb	
Hal13	(GT)_17_	147430	0	0	0		
Aa12	(GT)_12_	109279	0	0	0	Nearest gene within 302 kb	
Aa27	(CA)_11_	71173	0	0	0	Nearest gene within 72 kb	
Aa39	(AC)_13_	24219	0	0	0	Nearest gene within 40 kb	
IEAAAG04	(AAAG)_6_(AAAC)_4_(AAAG)_6_	134664	0	0	0	Nearest gene within 140 kb	
IEAAAG13	(AAAG)_3_(RAAG)_13_(AAAG)_16_	302707	0	0	0	Nearest gene within 77 kb	
IEAAAG14	(AAAG)_18_	363834	0	0	0	Nearest gene within 165 kb	
IEAAAG15	(AAAG)_7_	464074	1	1	1	Microsatellite within gene	Tumor necrosis factor

Our search for additional *A. chrysaetos* markers revealed 60,346 microsatellites (34,443 dinucleotides, 16,660 trinucleotides, 5,370 tetranucleotides, 3,389 pentanucleotides, and 484 hexanucleotides). We also identified 767,898 biallelic SNPs with read depths between 10–60x with quality scores greater than 20, which corresponds to 0.77 SNPs per Kbp.

The putative *A. chrysaetos* SWS1 opsin gene aligned with 100% identity to that of *Buteo buteo* and *Pandion haliaetus*, and with 99% identity to *Accipiter gentilis* (see supplementary material). The translated amino acid sequence (FISCIFSVFTV) indicates a violet-tuned color vision system as opposed to ultraviolet [55].

## Discussion

We have sequenced, assembled, and annotated the *A. chrysaetos* genome. Avian genomics is still in its infancy and thus meaningful comparisons of the eagle genome with other bird genomes are difficult. Extant birds are generally grouped into more than 200 families, yet complete genome sequences are currently restricted to 10 avian families and no other members of the family Accipitridae (Table 1). Avian genome assemblies range in size from 1.04 Gb in the Tibetan Ground-tit to 1.55 Gb in the Golden Eagle (Table 1). NCBI contains far more sequenced mammalian genomes (n>50), the assemblies of which are larger (mean of 2.5 Gb) and more variable in size (range 2.00 Gb to 4.21 Gb) than avian genomes. The homogeneity in avian genome size relative to mammalian genome size is also reflected in flow cytometry data [58]. *A. chrysaetos* gene lengths are similar to other birds but mean exon and intron lengths are somewhat shorter (Table 1), suggesting that promoters, 5′ UTRs, and 3′UTRs may be longer in eagles.

Golden eagle genome size estimates range from 1.28–1.48 Gb ([59], [60], Table 1), indicating that our assembly is potentially 5–21% larger than the actual genome size. Bradnam et al. [61] argued that large assemblies may result from assembly errors, but can also occur when heterogeneous regions of the genome are legitimately resolved into independent scaffolds. This study also provided evidence that assemblies which are relatively larger or smaller than the estimated genome size can perform well in terms of other metrics, such as the number of correctly identified core eukaryotic genes. The “completeness” of our overall genome assembly is indeed evidenced by our identification of most all (90%) core eukaryotic genes (CEGs; [29], Table S6 in File S1); as well as by our microsatellite mapping exercise (i.e., all 15 anonymous loci were identified in our scaffolds) and our recovery of the entire *A. chrysaetos* mtDNA genome sequence. These results are comparable to recently published, high-quality genomes (e.g., rock pigeon [50]) and indicate that our assembly includes the vast majority of *A. chrysaetos* genes.

Our xenobiotic analyses, whereby we parsed eagle (vertebrate) sequences from invertebrate sequences, revealed that blood from the propositus also contained DNA from other species. Thus, our deep sequencing identified previously uncharacterized organisms that may be important to the ecology and evolution of *A. chrysaetos*. For example, these xenobiotic sequences include hits to a number of avian retroviruses, viruses, and pathogenic bacteria (Table S3 in File S1).

The repertoire of repetitive DNA in *A. chrysaetos* is limited relative to mammals, but is generally similar to known avian genomes (Table S8 in File S1, [62], [63]). The *A. chrysaetos* genome does not exhibit substantial variation in repeat content, either in the total proportion of repeats in the genome or in the relative proportions of different superfamilies and/or classes of repetitive elements. The *A. chrysaetos* genome appears to have fewer LINEs than the chicken genome [64], but this could also be attributable to technical factors such as enrichment of repetitive regions in unassembled portions of the genome and/or incomplete repeat libraries (see supplementary material). Overall, the lack of variation in repeat contents is consistent with the relative homogeneity of avian genome sizes compared to mammalian genomes [62], [63].

We annotated 16,571 genes in the golden eagle genome, including orthologs, for example, to *Bmp4*, a gene implicated in raptor beak formation [42]. These annotations are the first step to exploring unique golden eagle adaptations. For example, 57 predicted genes have ontologies associated with olfaction (e.g., olfactory receptors), a number similar to saker and peregrine falcons. Historically, birds were thought to rely primarily on magnetic or visual cues to hunt and navigate. As a result, only a few studies have addressed avian sensitivity to and navigation by odor [65], [66] or the olfactory receptor (OR) genes that may underlie these abilities [42], [67]. Our identification of OR genes may ultimately allow scientists to determine the molecular mechanisms underlying eagle olfaction, which may be important in locating carrion in forests or fish in the open sea.

Genome sequencing provides opportunities to develop new tools for species of conservation concern. MtDNA has been used to quantify genetic variation of threatened species, identify evolutionary distinct populations, and evolutionary significant units [68], [69]. Molecular clock analyses based on the mtDNA genome sequence [see ESM] suggest the golden eagle diverged from the Mountain Hawk-Eagle roughly 2.1 MYA, and from the Peregrine Falcon roughly 4.6 years ago. These estimates are generally consistent with previously published molecular phylogenies [70]. Our estimate of overall nucleotide variability (0.77 SNPs per Kbp), is remarkably similar to estimates of SNP density of the scarlet macaw, saker and peregrine falcons (0.86, 0.63, and 0.88 SNPs per Kbp; respectively) but considerably less than the 1.75 SNPs per Kbp of zebra finch [36], [71], [72].

Our SWS1 opsin gene analysis provides evidence only for a vision system biased toward violet (VS) vision, rather than ultraviolet (UVS). Avian species with a VS-tuned vision are particularly sensitive at wavelengths above 400 nm, while UVS-tuned birds are sensitive at wavelengths below 400 nm [73], [74]. Although classic studies suggested that raptors hunt by following ultraviolet signals in the urine of prey [75], Odeen and Hastad [55] determined that VS-tuned systems are predominant in raptors. They additionally hypothesized that UVS-tuned passerine prey may be able to communicate with one another using colors inconspicuous to raptors. Furthermore, Lind et al. [73] measured transmission properties of tissues (ocular media transmittance) in the common buzzard eye and argued that the chromatic contrast between vole urine and substrate would provide an unreliable cue to hunting raptors. Taken in total, these results provide little evidence that golden eagles are sensitive to ultraviolet light, and thus that UV-reflective paint likely would not increase the visibility of structures and prevent golden eagle collisions.

Genome sequencing also provides geneticists with opportunities to investigate assumptions associated with previously-developed tools. For example, microsatellite markers are commonly used in studies of natural populations, but the vast majority of these markers are anonymous with respect to their position in the genome. Disequilibrium tests are often used to determine if microsatellites are inherited independently of one another, but such tests do not include genomic position. This may be important, as eukaryotic genomes are not homogenous and selection can vary greatly across the genome [76]. Microsatellites located in or near functional genes are likely to be more exposed to selection and selective sweeps than those occurring in gene deserts, and it is known that vertebrate microsatellites are often found in expressed genes [77].

Of Bourke and Dawson’s [51] 15 anonymous *A. chrysaetos* microsatellites, twelve were within 400 kb of an annotated gene and two were found in the intron or untranslated region of a gene. A published study [51] of over a hundred Scottish golden eagles found no deviations from Hardy-Weinberg expectations (HWE) at these twelve loci, but unpublished data on North American golden eagles found that seven of these twelve loci deviated from HWE (Maria Wheeler, personal communication). Hitchhiking is often suspected as the culprit when only one or a few microsatellite loci deviate from HWE in a population study, but as genome sequences become more commonplace, investigators will increasingly have the genomic infrastructure necessary to tease out location effects associated with functional genes.

Non-invasive molecular methods have the capacity to profoundly influence our understanding of threatened and endangered species [12], [13], [78]–[81]. For example, DNA fingerprints associated with naturally shed feathers have provided estimates of population size, reproductive success, and demographic turnover in Imperial Eagles (*A. heliaca,* [12], [13]). Genomic resources such as those reported herein will help extend studies based on anonymous genetic markers to those that include important functional genes. These might include avian genes associated with migratory tendencies, beak development, and olfaction [36], [71]. Future study of these (and other) genes will no doubt reveal their functional, molecular contributions to the widespread distribution of *A. chrysaetos* and their trophic position as apex predators. Thus, we anticipate that the *A. chrysaetos* genome sequence will guide our understanding of avian adaptation, while providing additional molecular tools that facilitate the conservation of these charismatic organisms.

## Supporting Information

Figure S1 **The Mexican coat of arms contains a golden eagle.**
(TIF)

Figure S2 **17 bp-mer estimation of the genome size of** ***A. chrysaetos*** **.** K-mer depth is on the x-axis, while the frequency of K-mer counts at a given sequencing depth is represented on the y-axis.
(TIF)

File S1 **Table S1, Table S2, Table S3, Table S4, Table S5, Table S6, Table S7, Table S8, Table S9 and Table S10. Table S1.** Software used to assemble, annotate, and describe the *A. chrysaetos* genome. ORF, open reading frame. **Table S2.** 70-mer statistics for the *Aquila chrysaetos* genome. **Table S3.** Summary of the BLASTN search against NCBI nucleotide database (BLASTN parameters: E 697 value = 1E-6, 1000 hits per each query). The contigs with only non-vertebrate hits are listed along with 698 the description of hits. When the BLASTN search resulted in >3 hits from the same group (indicated by 699*), only the top 3 hits for each taxonomic group are listed. **Table S4.** *Aquila chrysaetos* genome data production. **Table S5.** Mitochondrial gene profile of *Aquila chrysaetos.* **Table S6.** Identification of CEGs (partial and complete) in the *Aquila chrysaetos* genome. **Table S7.** Top Pfam domain hits and their counts. **Table S8.** Repetitive elements expressed as percentages of avian genomes. Note that comparisons among assemblies are complicated by technical differences in genome assembly and databases employed. **Table S9.** Bourke and Dawson’s microsatellites, their reported sizes in [51], and observed size in the *A.* 500 *chrysaetos* genome assembly. **Table S10.** Genomic composition of avian mitochondrial DNA.
(DOCX)
