# Supplementary figures and images for: The Genome Sequence of a Widespread Apex Predator, the Golden Eagle (Aquila chrysaetos)
Source: PLoS One. 2014 Apr 23;9(4):e95599. doi: 10.1371/journal.pone.0095599 (PMC3997482; doi:10.1371/journal.pone.0095599)

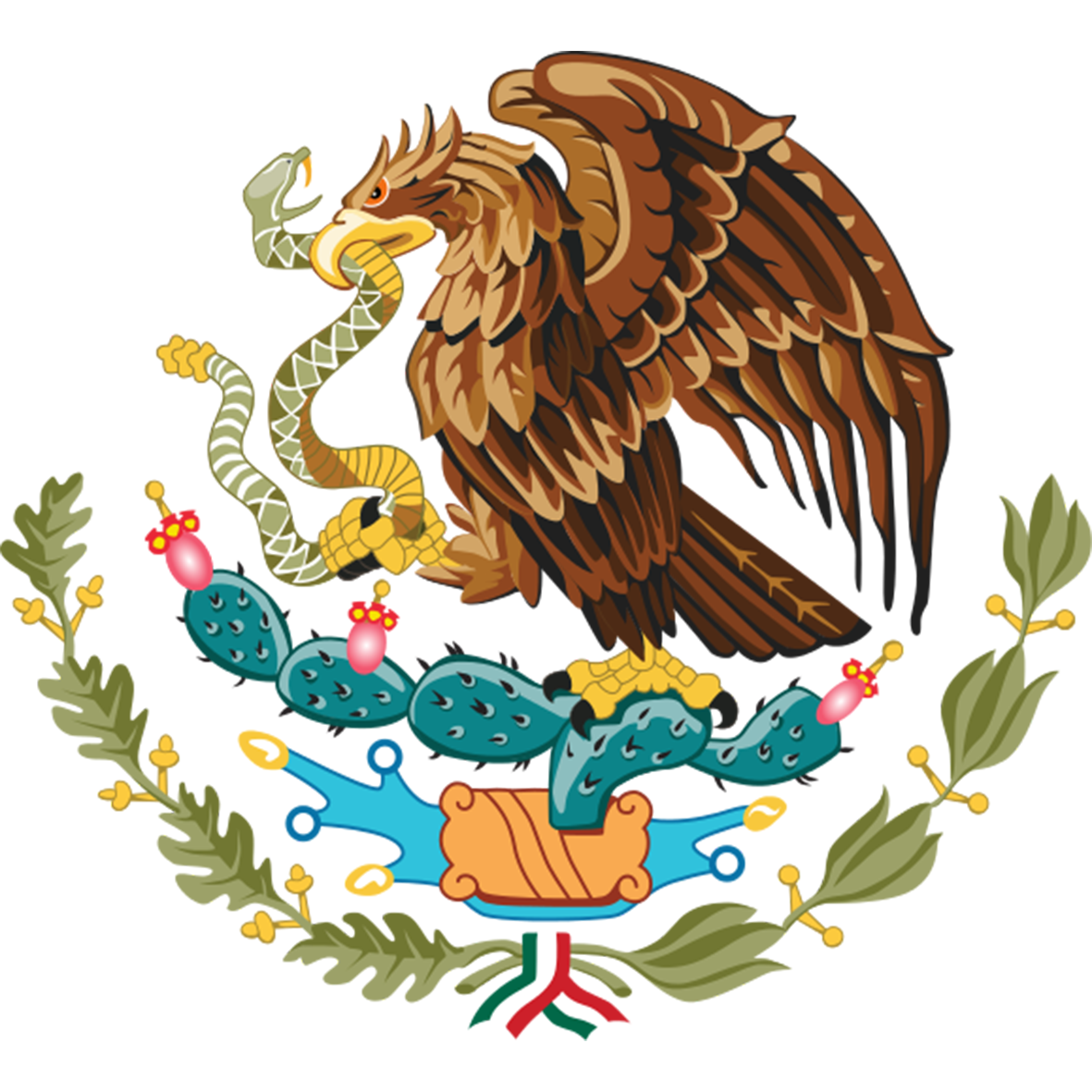

Supplement: Figure S1 — The Mexican coat of arms contains a golden eagle. (TIF) [file pone.0095599.s001.tif]

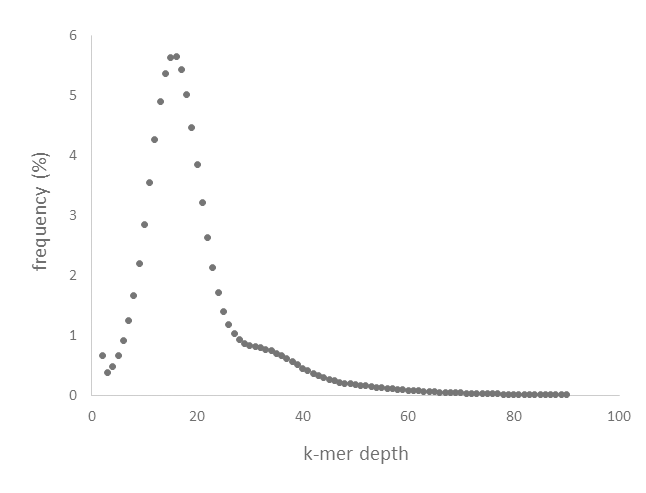

Supplement: Figure S2 — 17 bp-mer estimation of the genome size of A. chrysaetos . K-mer depth is on the x-axis, while the frequency of K-mer counts at a given sequencing depth is represented on the y-axis. (TIF) [file pone.0095599.s002.tif]
