# Supplementary material for: The Genome Sequence of a Widespread Apex Predator, the Golden Eagle (Aquila chrysaetos)
Source: PLoS One. 2014 Apr 23;9(4):e95599. doi: 10.1371/journal.pone.0095599 (PMC3997482; doi:10.1371/journal.pone.0095599)
Supplement: File S1 — Table S1, Table S2, Table S3, Table S4, Table S5, Table S6, Table S7, Table S8, Table S9 and Table S10. Table S1. Software used to assemble, annotate, and describe the A. chrysaetos genome. ORF, open reading frame. Table S2. 70-mer statistics for the Aquila chrysaetos genome. Table S3. Summary of the BLASTN search against NCBI nucleotide database (BLASTN parameters: E 697 value = 1E-6, 1000 hits per each query). The contigs with only non-vertebrate hits are listed along with 698 the description of hits. When the BLASTN search resulted in >3 hits from the same group (indicated by 699*), only the top 3 hits for each taxonomic group are listed. Table S4. Aquila chrysaetos genome data production. Table S5. Mitochondrial gene profile of Aquila chrysaetos. Table S6. Identification of CEGs (partial and complete) in the Aquila chrysaetos genome. Table S7. Top Pfam domain hits and their counts. Table S8. Repetitive elements expressed as percentages of avian genomes. Note that comparisons among assemblies are complicated by technical differences in genome assembly and databases employed. Table S9. Bourke and Dawson’s microsatellites, their reported sizes in [51], and observed size in the A. 500 chrysaetos genome assembly. Table S10. Genomic composition of avian mitochondrial DNA. (DOCX) [file pone.0095599.s003.docx]

***Electronic Supplementary Materials for:***

**The genome sequence of a widespread apex predator, the golden eagle (*Aquila chrysaetos*)**

**_____________________________________________________________________________________**

**Jacqueline M. Doyle^1^, Todd E. Katzner^3,4^, Peter H. Bloom^5^, Yanzhu Ji^1^, Bhagya K. Wijayawardena^1,2^ and J. Andrew DeWoody^1,2^**

**_____________________________________________________________________________________**

**^1^Departments of Forestry & Natural Resources and ^2^Biological Sciences, Purdue University, West Lafayette IN 47907**

**^3^Division of Forestry & Natural Resources, West Virginia University, Morgantown WV 26506**

**^4^Northern Research Station, USDA Forest Service, Parsons WV 26287**

**^5^Western Foundation of Vertebrate Zoology, 439 Calle San Pablo, Camarillo CA 93012**

**Corresponding authors: Jacqueline Doyle (**[**jmdoyle@purdue.edu**](file:///C:\Users\dewoody\AppData\Local\Microsoft\Windows\Temporary%20Internet%20Files\Content.Outlook\54QZL6M9\jmdoyle@purdue.edu)**)**

**(a) Sampling, molecular methods, and quality control**

Following capture, the male golden eagle was subsequently tracked using a GPS-GSM telemetry unit manufactured by Cellular Tracking Technologies, LLC. Between capture in December 2012 and March 2013, the eagle had a home range (95% KDE) of approximately 1,000km^2^.

The paired-end genomic library was composed of sequences with a mean insert size of 366 bp, whereas the mate-paired genomic library contained inserts with a mean size of 1.4 Kb. We conducted a *de novo* assembly of the golden eagle nuclear genome because at the time of writing, no reference genomes of close relatives were available in public databases.

**(b) Genome assembly and genome size estimation**

The golden eagle genome was assembled in ABySS with the following parameters: abyss-pe s=202 n=10 k=70  l=30; followed by file specifications for 1) 'lib' -- the paired-end and mate-pair reads, 2) 'se'  -- the unpaired and mate-pair reads used as single-end reads in assembly and 3) 'mp'  -- the paired-end and mate-pair reads used in scaffolding.

We estimated the actual coverage of the genome (N) using the equation N = M/((L-K+1)/L), when L is the mean read length, K is the kmer size and M is the mean K-mer coverage [1]. Peak depth (M) was equal to 16 (Figure S1) and genome coverage (N) was equal to 19.1. We divided our total sequence data (24,385,716,189 bp) by N to calculate a genome size of 1,276,739,067 bp.

We used two processes to assemble the *A. chrysaetos* mitochondrial genome. First, we assembled our paired-end reads to the mountain hawk-eagle mitochondrial genome [2] using Bowtie2 [2] with the very-fast-local setting. Second, we used the program MITObim [3], which uses a baiting-and-iterative mapping approach, to reconstruct the golden eagle mitochondrial genome. To initiate MITObim [3], we seeded the reconstruction with an *A. chrysaetos* mitochondrial barcode sequence (GU571265). Reads were subsequently mapped to the reference sequence, and the length of the reconstructed mitochondrial sequence was extended with each iteration. The process ended when the reconstructed sequence could not be extended any farther (in our case after 122 iterations). In the case of the Bowtie2-produced genome only, 457 bases could not be resolved. We estimated the divergence time between the golden eagle and the Mountain Hawk Eagle and the Peregrine falcon using the equation T = d/2r; where r = 0.021 [4] and d = 0.089 and 0.193 for the Mountain Hawk Eagle and Peregrine Falcon, respectively.

**(c) Gene annotation**

We used DOGMA [4] to annotate the mitochondrial genome (parameter settings: gapped alignment, 60% identity cutoff for protein coding genes, 55% identity cutoff for RNAs, e-value < 1e-5, COVE threshold for mt tRNAs of 7) and OGDraw [5] to draw the mitochondrial gene map. The putative control region was identified based on 92% similarity with the *S. nipalensis* control region and 99% similarity with an *A. chrysaetos* partial control region sequence (EF459579). Fourteen tRNA genes were found on the α-strand of the mtDNA molecule and the remaining nine tRNA genes on the β-strand. *A. chrysaetos*, like many avian species, also has mitochondrial protein-coding and rRNA genes with slight A+T biases, while a more exaggerated A+T bias can be found in the control region (Table S10).

**(d) Xenobiotics and repetitive sequences**

Xenobiotic sequences were removed from the assembly before submission to NCBI. To facilitate direct comparison of repeat contents with peregrine and saker genomes [6], parameters in RepeatMasker [7] and RepeatProteinMask [7] were chosen based on Zhan et al. (2013) paper. Specifically, excluding xenobiotic scaffolds, we ran RepeatMasker with library option (‘-lib’) to include all repeats in Repbase [8] and provided with RepeatMasker version 4.0.2. All other parameters were set to default. RepeatProteinMask was run with E-value threshold of 1e-4, and all other parameters set to default. We additionally used the non-homology-based approach RepeatModeler with default parameters. Tandem repeats were detected using TRF [9] with default settings. Results from RepeatMasker, RepeatProteinMask, non-homology-based approaches, and TRF were combined to remove overlapping regions and calculate overall repeat content using a custom perl script (modified from L. Hu, personal communication).

**(e) Linkage disequilibrium and molecular markers**

We identified microsatellite markers using the program MISA. We searched for dinucleotides with a minimum of 6 repeats and for trinucleotides, tetranucleotides, pentanucleotides and hexanucleotides with a minimum of 5 repeats.

**(f) Color vision determination**

The *A. chrysaetos* SWS1 opsin nucleotide and amino acid sequences are as follows: AAGACGGTGAAGACGCTGAAGATGCAGGAGATGAAGCCGCTGAAGGAGATGTTCACCAAG and FISCIFSVFTV.

References

1. Li R, Fan W, Tian G, Zhu H, He L, et al. (2010) The sequence and de novo assembly of the giant panda genome. Nature 463: 311–317. Available: http://www.ncbi.nlm.nih.gov/pubmed/20010809. Accessed 7 August 2013.

2. Langmead B, Salzberg SL (2012) Fast gapped-read alignment with Bowtie 2. Nat Methods 9: 357–359. Available: http://www.pubmedcentral.nih.gov/articlerender.fcgi?artid=3322381&tool=pmcentrez&rendertype=abstract. Accessed 6 August 2013.

3. Hahn C, Bachmann L, Chevreux B (2013) Reconstructing mitochondrial genomes directly from genomic next-generation sequencing reads--a baiting and iterative mapping approach. Nucleic Acids Res 41: e129. Available: http://www.pubmedcentral.nih.gov/articlerender.fcgi?artid=3711436&tool=pmcentrez&rendertype=abstract. Accessed 6 August 2013.

4. Wyman S, Jansen R, Boore J (2004) Automatic annotation of organellar genomes with DOGMA. Bioinformatics 20: 3252–3255.

5. Lohse M, Drechsel O, Kahlau S, Bock R (2013) OrganellarGenomeDRAW--a suite of tools for generating physica maps of plastid and mitochondrial genomes and visualizing expression data sets. Nucleic Acids Res.

6. Zhan X, Pan S, Wang J, Dixon A, He J, et al. (2013) Peregrine and saker falcon genome sequences provide insights into evolution of a predatory lifestyle. Nat Genet 45: 536–566. doi:10.1038/ng.2588.

7. Smit A, Hubley R, Green P (n.d.) RepeatMasker Open-3.0: http://www.repeatmasker.org.

8. Jurka J, Kapitonov V, Pavlicek A, Klonowski P, Kohany O, et al. (2005) Repbase update, a database of eukaryotic repetitive elements. Cytogenet Genome Res 110: 462–467.

9. Benson G (1999) Tandem repeats finder: a program to analyze DNA sequences. Nucleic Acids Res 27: 573–580.

10. Lohse M, Bolger A, Nagel A, Fernie A, Lunn J, et al. (2012) RobiNA: a user-friendly, integrated software solution for RNA-Seq-based transcriptomics. Nucleic Acids Res 40: W622–W627.

11. Simpson JT, Wong K, Jackman SD, Schein JE, Jones SJM, et al. (2009) ABySS: a parallel assembler for short read sequence data. Genome Res 19: 1117–1123. Available: http://www.pubmedcentral.nih.gov/articlerender.fcgi?artid=2694472&tool=pmcentrez&rendertype=abstract. Accessed 6 August 2013.

12. Parra G, Bradnam K, Ning Z, Keane T, Korf I (2009) Assessing the gene space in draft genomes. Nucleic Acids Res 37: 298–297.

13. Marçais G, Kingsford C (2011) A fast, lock-free approach for efficient parallel counting of occurrences of k-mers. Bioinformatics 27: 764–770. Available: http://www.pubmedcentral.nih.gov/articlerender.fcgi?artid=3051319&tool=pmcentrez&rendertype=abstract. Accessed 7 August 2013.

14. Grabherr M, Haas B, Yassour M, Levin J, Thompson D, et al. (2013) Trinity: reconstructing a full-length transcriptome without a genome from RNA-Seq data. Nat Biotechnol 29: 644–652. doi:10.1038/nbt.1883.Trinity.

15. Cantarel BL, Korf I, Robb SMC, Parra G, Ross E, et al. (2008) MAKER: an easy-to-use annotation pipeline designed for emerging model organism genomes. Genome Res 18: 188–196. Available: http://www.pubmedcentral.nih.gov/articlerender.fcgi?artid=2134774&tool=pmcentrez&rendertype=abstract. Accessed 7 August 2013.

16. Korf I (2004) Gene finding in novel genomes. BMC Bioinformatics 5: 59. Available: http://www.pubmedcentral.nih.gov/articlerender.fcgi?artid=421630&tool=pmcentrez&rendertype=abstract.

17. Stanke M, Waack S (2003) Gene prediction with a hidden Markov model and a new intron submodel. Bioinformatics 19: ii215–ii225. Available: http://bioinformatics.oxfordjournals.org/cgi/doi/10.1093/bioinformatics/btg1080. Accessed 20 September 2013.

18. Smit A, Hubley R (n.d.) RepeatModeler Open-1.0: http://www.repeatmasker.org.

19. Thiel T (2002) MISA - Microsatellite identification tool: http://pgrc.ipk–gatersleben.de/misa/.

20. Li H, Handsaker B, Wysoker A, Fennell T, Ruan J, et al. (2009) The sequence alignment/map (SAM) format and SAMtools. Bioinformatics 25: 2078–2079.

21. Lewis SE, Searle SMJ, Harris N, Gibson M, Lyer V, et al. (2002) Apollo: a sequence annotation editor. Genome Biol 3: 1–14. Available: http://www.pubmedcentral.nih.gov/articlerender.fcgi?artid=151184&tool=pmcentrez&rendertype=abstract.

Table S1: Software used to assemble, annotate, and describe the *A. chrysaetos* genome.

| Program | Citation | Purpose |
| --- | --- | --- |
| Trimmomatic vs. 0.30 | [10] | Read filtering |
| ABySS vs. 1.3.6 | [11] | *de novo* genome assembly |
| CEGMA vs. 2.4.010312 | [12] | Identifying core eukaryotic genes |
| Jellyfish vs. 1.1.6 | [13] | K-mer counting |
| Bowtie2 vs. 2.1.0 | [2] | Align sequence reads to reference sequences |
| MITObim vs. 1.5 | [3] | Mitochondrial genome assembly |
| DOGMA | [4] | Mitochondrial genome annotation |
| OGDraw | [5] | Mitochondrial genome visualization |
| Trinity vs. 2013_08_14 | [14] | *de novo* transcriptome assembly |
| MAKER vs. 2.28 | [15] | Gene annotation pipeline |
| RepeatMasker vs. 4.0.2 | [7] | Identify repetitive elements |
| SNAP vs. 2013_02-16 | [16] | *ab initio* gene prediction |
| AUGUSTUS vs. 2.7 | [17] | *ab initio* gene prediction |
| RepeatProteinMask vs. 4.0.2 | [7] | Identify ORFs^1^ in repetitive elements |
| RepeatModeler 1.0.7 | [18] | *de novo* prediction of repetitive elements |
| Tandem Repeats Finder vs. 4.04 | [9] | Identify tandem repeats |
| MISA | [19] | Identify microsatellite markers |
| Samtools 1.18 | [20] | SNP prediction |
| Apollo vs. 1.11.8 | [21] | Genome annotation viewer and editor |

^1^ORF, open reading frame

Table S2: 70-mer statistics for the *Aquila chrysaetos* genome

| Scaffolds | Number of scaffolds | Total (b) | Range (b) | N50 (b) |
| --- | --- | --- | --- | --- |
| > 200 bp | 42,926 | 1,548,489,457 | 200-11,517,212 | 1,746,960 |
| > 10000 bp | 2,552 | 1,524,561,899 | 10002-11,517,212 | 1,765,888 |
| > 1200000 bp | 415 | 1,005,754,426 | 1205009-11,517,212 | 2,544,707 |

Table S3: Summary of the BLASTN search against NCBI nucleotide database (BLASTN parameters: E value= 1E-6, 1000 hits per each query). The contigs with only non-vertebrate hits are listed along with the description of hits. When the BLASTN search resulted in >3 hits from the same group (indicated by *), only the top 3 hits for each taxonomic group are listed.

| Viruses | | Bacteria | Protists | Fungi | | | Insects | Planaria | Plants | Chordates |  |
| --- | --- | --- | --- | --- | --- | --- | --- | --- | --- | --- | --- |
| Golden eagle contig ID | | | | Contig length | BLAST hit description | | | | | | E-value/ %ID/ Alignment length in bp |
| kmer70-min200.2250482 | | | | 1959 | *Branchiostoma floridae* hypothetical protein, mRNA | | | | | | 3E-14/ 83.12/ 77 |
| kmer70-min200.2283777 * | | | | 885 | *Helicobacter pylori* GAMchJs117Ai H_pylori-1.0_Cont5.1, whole genome shotgun sequence | | | | | | 0/ 100/ 587 |
|  |  |  |  |  | *Helicobacter pylori* GAMchJs114i H_pylori-1.0_Cont10.1, whole genome shotgun sequence | | | | | | 0/ 100/ 587 |
|  |  |  |  |  | *Enterobacteria* phage phiX174 isolate XC+MbD22ic7, complete genome | | | | | | 0/ 99.83/ 587 |
|  |  |  |  |  | *Enterobacteria* phage phiX174 isolate XC+mbD10im2, complete genome | | | | | | 0/ 99.83/ 587 |
|  |  |  |  |  | *Enterobacteria* phage phiX174 isolate XC+Mbd10im1, complete genome | | | | | | 0/ 99.83/ 587 |
| kmer70-min200.2284383 * | | | | 655 | *Helicobacter pylori* GAMchJs117Ai H_pylori-1.0_Cont5.1, whole genome shotgun sequence | | | | | | 3E-154/ 100/ 300 |
|  |  |  |  |  | *Helicobacter pylori* GAMchJs114i H_pylori-1.0_Cont10.1, whole genome shotgun sequence | | | | | | 3E-154/ 100/ 300 |
|  |  |  |  |  | Synthetic *Enterobacteria* phage phiX174.1f, complete genome | | | | | | 3E-154/ 100/ 300 |
|  |  |  |  |  | *Enterobacteria* phage phiX174 strain beta4, complete genome | | | | | | 3E-154/ 100/ 300 |
|  |  |  |  |  | *Enterobacteria* phage phiX174 strain beta3, complete genome | | | | | | 3E-154/ 100/ 300 |
| kmer70-min200.185194* | | | | 349 | *Helicobacter pylori* GAMchJs117Ai H_pylori-1.0_Cont5.1, whole genome shotgun sequence | | | | | | 0/ 100/ 349 |
|  |  |  |  |  | *Helicobacter pylori* GAMchJs114i H_pylori-1.0_Cont10.1, whole genome shotgun sequence | | | | | | 0/ 100/ 349 |
|  |  |  |  |  | Mutant *Enterobacteria* phage phiX174 isolate PhiX_10_3, complete genome | | | | | | 0/ 100/ 349 |
|  |  |  |  |  | Mutant *Enterobacteria* phage phiX174 isolate PhiX_10_4, complete genome | | | | | | 0/ 100/ 349 |
|  |  |  |  |  | Mutant *Enterobacteria* phage phiX174 isolate PhiX_10_2, complete genome | | | | | | 0/ 100/ 349 |
| kmer70-min200.443522 | | | | 311 | *Strobopagurus gracilipes* voucher NTOU A00009 phosphoenolpyruvate carboxykinase (PEPCK) gene, partial cds | | | | | | 1E-14/ 90.91/ 66 |
|  |  |  |  |  | *Eduarctus martensii* phosphoenolpyruvate carboxykinase (PEPCK) gene, partial cds | | | | | | 5E-09/ 97.56/ 46 |
| kmer70-min200.566924 | | | | 292 | *Columba palumbus* retrovirus partial pol and pro genes isolate RV-Wood Pigeon | | | | | | 2E-12/ 74.15/ 205 |
| kmer70-min200.1246121 | | | | 268 | HIV-1 isolate 69-6 from Australia pol polyprotein (pol) gene, partial cds | | | | | | 4E-09/ 93.62/ 47 |
| kmer70-min200.1401212 | | | | 248 | *Acanthamoeba castellanii* str. Neff hypothetical protein (ACA1_113910) mRNA, complete cds | | | | | | 2E-07/ 91.49/ 47 |
| kmer70-min200.1080684* | | | | 233 | *Helicobacter pylori* GAMchJs117Ai H_pylori-1.0_Cont5.1, whole genome shotgun sequence | | | | | | 1E-117/ 100/ 233 |
|  |  |  |  |  | *Helicobacter pylori* GAMchJs114i H_pylori-1.0_Cont10.1, whole genome shotgun sequence | | | | | | 1E-117/ 100/ 233 |
|  |  |  |  |  | Synthetic *Enterobacteria* phage phiX174.1f, complete genome | | | | | | 1E-117/ 100/ 233 |
|  |  |  |  |  | Mutant *Enterobacteria* phage phiX174 isolate PhiX_10_3, complete genome | | | | | | 1E-117/ 100/ 233 |
|  |  |  |  |  | Mutant *Enterobacteria* phage phiX174 isolate PhiX_10_5, complete genome | | | | | | 1E-117/ 100/ 233 |
| kmer70-min200.853467 | | | | 233 | *Anopheles gambiae* str. PEST AGAP003350-PA (AgaP_AGAP003350) mRNA, complete cds | | | | | | 6E-07/ 95.12/ 41 |
|  |  |  |  |  | *Anopheles gambiae* str. PEST AGAP003350-PB (AgaP_AGAP003350) mRNA, complete cds | | | | | | 9E-07/ 95.12/ 41 |
| kmer70-min200.301194 | | | | 218 | *Columba palumbus* retrovirus partial pol and pro genes isolate RV-Wood Pigeon | | | | | | 6E-12/ 74.49/ 196 |
| kmer70-min200.1836992* | | | | 215 | *Zea mays* clone 1629683 mRNA sequence | | | | | | 9E-10/ 80.77/ 104 |
|  |  |  |  |  | *Zea mays* full-length cDNA clone ZM_BFb0299P06 mRNA, complete cds | | | | | | 3E-09/ 80.81/ 99 |
|  |  |  |  |  | *Zea mays* clone 258380 glycine-rich RNA-binding protein 2 mRNA, complete cds | | | | | | 3E-09/ 80.81/ 99 |

Table S4: *Aquila chrysaetos* genome data production

|  |  | Raw data | | After filtering | |
| --- | --- | --- | --- | --- | --- |
| Library | Read length (bp) | Total data (Gb) | Sequence coverage (X) | Total data (Gb) | Sequence coverage (X) |
| Paired-end | 100 | 25.3 | 19.6 | 24.5 | 19.1 |
| Mate-paired | 100 | 43.1 | 33.4 | 21.0 | 19.8 |
| Total |  | 68.4 | 53.0 | 45.5 | 38.9 |

Table S5: Mitochondrial gene profile of *Aquila chrysaetos*

| MITObim assembly | | | | Mapping with Bowtie2 to *Spizaetus nipalensis* | | | |
| --- | --- | --- | --- | --- | --- | --- | --- |
| Feature | Position number | | Size (bp) | Feature | Position number | | Size (bp) |
|  | From | To |  |  | From | To |  |
| Cob | 7 | 1,146 | 1,139 | Cob | 1 | 1,140 | 1,139 |
| trnT-ugu | 1,154 | 1,223 | 69 | trnT-ugu | 1,146 | 1,214 | 68 |
| trnP-ugg | (2,382 | 2,451)^1^ | 69 | trnP-ugg | (2,373 | 2,442) | 69 |
| Nad6 | (2,468 | 2,983) | 515 | Nad6 | (2,458 | 2,973) | 515 |
| trnE-uuc | (2,987 | 3,057) | 70 | trnE-uuc | (2,977 | 3,047) | 70 |
| trnF-gaa | 3,685 | 3,748 | 63 | trnF-gaa | 4,007 | 4,076 | 69 |
| rrnS | 3,755 | 4,722 | 967 | rrnS | 4,077 | 5,043 | 966 |
| trnV-uac | 4,725 | 4,796 | 71 | trnV-uac | 5,046 | 5,117 | 71 |
| rrnL | 4,839 | 6,375 | 1,536 | rrnL | 5,127 | 6,699 | 1,572 |
| trnL-uaa | 6,385 | 6,458 | 73 | trnL-uaa | 6,715 | 6,788 | 73 |
| Nad1 | 6,468 | 7,442 | 974 | Nad1 | 6,798 | 7,757 | 959 |
| trnI-gau | 7,444 | 7,515 | 71 | trnI-gau | 7,773 | 7,844 | 71 |
| trnQ-uug | (7,529 | 7,599) | 70 | trnQ-uug | (7,858 | 7,928) | 70 |
| trnM-cau | 7,599 | 7,667 | 68 | trnM-cau | 7,928 | 7,996 | 68 |
| Nad2 | 7,668 | 8,696 | 1,028 | Nad2 | 7,997 | 9,025 | 1,028 |
| trnS-uga | (8,706 | 8,777) | 71 | trnS-uga | (9,035 | 9,106) | 71 |
| trnW-uca | 8,707 | 8,778 | 71 | trnW-uca | 9,036 | 9,107 | 71 |
| trnA-ugc | (8,780 | 8,848) | 68 | trnA-ugc | (9,109 | 9,177) | 68 |
| trnN-guu | (8,851 | 8,923) | 72 | trnN-guu | (9,181 | 9,253) | 72 |
| trnC-gca | (8,926 | 8,992) | 66 | trnC-gca | (9,256 | 9,322) | 66 |
| trnY-gua | (8,992 | 9,062) | 70 | trnY-gua | (9,322 | 9,392) | 70 |
| Cox1 | 9,064 | 10,611 | 1,547 | Cox1 | 9,394 | 10,941 | 1,547 |
| trnS-uga | (10,606 | 10,679) | 73 | trnS-uga | (10,936 | 11,009) | 73 |
| trnD-guc | 10,685 | 10,753 | 68 | trnD-guc | 11,015 | 11,083 | 68 |
| Cox2 | 10,756 | 11,436 | 680 | Cox2 | 11,086 | 11,766 | 680 |
| trnL-aag | 11,441 | 11,511 | 70 | trnL-aag | 11,771 | 11,840 | 69 |
| Atp8 | 11,513 | 11,674 | 161 | Atp8 | 11,842 | 12,006 | 164 |
| Atp6 | 11,671 | 12,351 | 680 | Atp6 | 12,000 | 12,680 | 680 |
| Cox3 | 12,354 | 13,136 | 782 | Cox3 | 12,683 | 13,465 | 782 |
| trnG-ucc | 13,138 | 13,206 | 68 | trnG-ucc | 13,467 | 13,535 | 68 |
| Nad3 | 13,207 | 13,554 | 348 | Nad3 | 13,536 | 13,883 | 347 |
| trnR-ucg | 13,565 | 13,633 | 68 | trnR-ucg | 13,894 | 13,962 | 68 |
| Nad4L | 13,635 | 13,928 | 293 | Nad4L | 13,964 | 14,257 | 293 |
| Nad4 | 13,925 | 15,301 | 1,376 | Nad4 | 14,254 | 15,630 | 1376 |
| trnH-gug | 15,303 | 15,372 | 69 | trnH-gug | 15,632 | 15,701 | 69 |
| trnS-gcu | 15,373 | 15,438 | 65 | trnS-gcu | 15,702 | 15,767 | 65 |
| trnL-uag | 15,439 | 15,509 | 70 | trnL-uag | 15,768 | 15,838 | 70 |
| Nad5 | 15,510 | 17,324 | 1,814 | Nad5 | 15,839 | 17,647 | 1,808 |

^1^ Brackets denote β-strand transcriptional polarity

Table S6: Identification of CEGs (partial and complete) in the *Aquila chrysaetos* genome

| CEG Group | # in assembly | % of group |
| --- | --- | --- |
| 1 | 60 | 90.9 |
| 2 | 53 | 94.6 |
| 3 | 56 | 91.8 |
| 4 | 55 | 84.6 |
| All | 224 | 90.3 |

Table S7: Top Pfam domain hits and their counts

| Pfam domain | Counts |
| --- | --- |
| WD domain, G-beta repeat | 643 |
| Immunoglobulin I-set domain | 564 |
| Fibronectin type III domain | 516 |
| Cadherin domain | 438 |
| Protein kinase domain | 375 |
| Collagen triple helix repeat (20 copies) | 364 |
| Zinc-finger double domain | 310 |
| 7 transmembrane receptor (rhodopsin family) | 303 |
| Ankyrin repeats (3 copies) | 301 |
| Leucine rich repeat | 285 |
| RNA recognition motif. (a.k.a. RRM, RBD, or RNP domain) | 213 |
| Calcium-binding EGF domain | 206 |
| Kelch motif | 200 |
| Sushi domain (SCR repeat) | 195 |
| PDZ domain (Also known as DHR or GLGF) | 192 |
| Immunoglobulin domain | 174 |
| C2 domain | 174 |
| Zinc finger, C2H2 type | 171 |
| C2H2-type zinc finger | 166 |
| EGF-like domain | 165 |
| Ion transport protein | 162 |
| BTB/POZ domain | 156 |
| Low-density lipoprotein receptor domain class A | 152 |
| Spectrin repeat | 152 |
| Tetratricopeptide repeat | 138 |
| Ras family | 135 |
| Thrombospondin type 1 domain | 135 |
| Homeobox domain | 134 |
| CUB domain | 130 |
| Immunoglobulin V-set domain | 126 |
| PH domain | 123 |
| Laminin EGF-like (Domains III and V) | 123 |
| LIM domain | 114 |
| Variant SH3 domain | 109 |
| Leucine Rich repeat | 106 |
| EF-hand domain pair | 103 |
| Laminin G domain | 96 |
| Mitochondrial carrier protein | 92 |
| Protein tyrosine kinase | 90 |
| Armadillo/beta-catenin-like repeat | 90 |
|  |  |
|  |  |
| Pfam domain | Counts |
| Ankyrin repeat | 89 |
| Scavenger receptor cysteine-rich domain | 85 |
| TPR repeat | 83 |
| Myosin head (motor domain) | 82 |
| Leucine Rich Repeat | 81 |
| Helicase conserved C-terminal domain | 81 |
| SAM domain (Sterile alpha motif) | 81 |
| Trypsin | 81 |
| von Willebrand factor type A domain | 80 |
| RhoGAP domain | 79 |
| Zinc finger, C3HC4 type (RING finger) | 78 |
| SH2 domain | 73 |
| Low-density lipoprotein receptor repeat class B | 72 |
| SH3 domain | 71 |
| Nebulin repeat | 70 |
| Helix-loop-helix DNA-binding domain | 70 |
| Cytochrome P450 | 69 |
| Plectin repeat | 69 |
| Calponin homology (CH) domain | 69 |
| Lectin C-type domain | 68 |
| Intermediate filament protein | 68 |
| Protein-tyrosine phosphatase | 67 |
| Coagulation Factor Xa inhibitory site | 66 |
| KH domain | 65 |
| IQ calmodulin-binding motif | 65 |
| RhoGEF domain | 63 |
| ABC transporter | 63 |
| Ubiquitin carboxyl-terminal hydrolase | 60 |
| Olfactory receptor | 55 |
| SPRY domain | 54 |
| Regulator of chromosome condensation (RCC1) repeat | 54 |
| von Willebrand factor type D domain | 53 |
| BTB And C-terminal Kelch | 52 |
| Ring finger domain | 51 |
| Kazal-type serine protease inhibitor domain | 51 |
| Neurotransmitter-gated ion-channel transmembrane region | 51 |
| Major Facilitator Superfamily | 50 |
| MORN repeat | 49 |
| Receptor family ligand binding region | 48 |
| PHD-finger | 48 |
| ATPase family associated with various cellular activities (AAA) | 48 |
| Pfam domain | Counts |
| short chain dehydrogenase | 47 |
| Ligand-binding domain of nuclear hormone receptor | 46 |
| WW domain | 45 |
| Kinesin motor domain | 45 |
| Human growth factor-like EGF | 44 |
| Neurotransmitter-gated ion-channel ligand binding domain | 44 |
| Bromodomain | 43 |
| 7 transmembrane receptor (Secretin family) | 43 |
| AAA domain | 43 |
| Hemopexin | 42 |
| Sugar (and other) transporter | 41 |
| AMP-binding enzyme | 40 |
| HMG (high mobility group) box | 40 |
| FERM central domain | 40 |
| Leucine Rich repeats (2 copies) | 39 |
| PX domain | 39 |
| RNA recognition motif (a.k.a. RRM, RBD, or RNP domain) | 39 |
| EF hand | 39 |
|  |  |

Table S8. Repetitive elements expressed as percentages of avian genomes. Note that comparisons among assemblies are complicated by technical differences in genome assembly and databases employed.

| Species | Total | LINEs | SINEs | LTR-RTs | DNA transposons | Unknown | Simple repeats  /Satellites  /Low complexity | Citations |
| --- | --- | --- | --- | --- | --- | --- | --- | --- |
| ***Aquila chrysaetos* (Golden Eagle)** | 5.94 | 2.57 | 0.13 | 1.40 | 0.54 | 0.51 | 0.94 |  |
| *Anas platyrhynchos* (Mallard) | 5.85 | 4.11 | 0.12 | 1.09 | 0.21 | 0.33 | NA | [22] |
| *Ara macaeo* (Scarlet Macaw)* | 5.20 | 3.46 | 0.08 | 0.95 | 0.05 | 0.04 | 0.63 | [23] |
| *Falco peregrinus* (Peregrine Falcon)** | 4.53 | 2.99 | 0.11 | 1.19 | 0.22 | 0.05 | 0.94 | [24] |
| *Falco cherrug* (Saker Falcon)** | 4.42 | 2.92 | 0.11 | 1.16 | 0.21 | 0.05 | 1.05 | [24] |
| *Ficedula albicollis* (Collared Flycatcher) | 10.68 | 3.05 | 0.07 | 1.44 | 0.04 | NA | 6.02 | [25] |
| *Gallus gallus* (Chicken) | 9.4 | 6.4 | 0.1 | 1.3 | 0.8 | NA | 0.80 | [26] |
| *Geospiza fortis* (Medium Ground-finch) | 3.3-4.1 | NA | NA | NA | NA | NA | NA | [27] |
| *Meleagris gallopavo* (Turkey) | 7.63 | 4.81 | 0.00 | 0.51 | 0.65 | 0.98 | 0.77 | [28] |
| *Pseudopodoces humilis* (Tibetan Ground-tit) | 7.11 | 3.67 | 0.10 | 3.03 | 0.00 | 0.05 | NA | [29] |
| *Taeniopygia guttata* (Zebra Finch) | 7.72 | 3.73 | 0.06 | 3.92*** | 0.01 | NA | 1.49 | [30] |
| *Columbia livia* (Rock Pigeon) | 8.72 | NA | NA | NA | NA | NA | NA | [31] |

* Data collected from unscaffolded assemblies.

** Data collected from homology-based prediction (RepeatMasker and RepeatProteinMask).

*** LTR-RT proportion summed by “LTR/ERV1”, “LTR/ERVK” and “LTR/ERVL”

Table S9: Bourke and Dawson’s microsatellites, their reported sizes in [53], and observed size in the *A. chrysaetos* genome assembly.

| Locus | Repeat motif | Expected product size^1^ | Scaffold product size |
| --- | --- | --- | --- |
| Aa43 | (AC)_14_ | 108-114 | 112 |
| Aa15 | (CA)_13_ | 197-203 | 201 |
| Aa26 | (AC)_14_ | 139-151 | 150 |
| Hal10 | (CA)_12_ | 228-238 | 231 |
| IEAAAG09 | (RAAG)_18_ | 486-490 | 486 |
| Aa11 | (CA)_11_ | 249-269 | 247 |
| Aa36 | (AC)_16_ | 92-124 | 102 |
| Hal13 | (GT)_17_ | 150-154 | 153 |
| Aa12 | (GT)_12_ | 150-162 | 153 |
| Aa27 | (CA)_11_ | 84-93 | 95 |
| Aa39 | (AC)_13_ | 184-192 | 187 |
| IEAAAG04 | (AAAG)_6_(AAAC)_4_(AAAG)_6_ | 228-244 | 231 |
| IEAAAG13 | (AAAG)_3_(RAAG)_13_(AAAG)_16_ | 241-249 | 246 |
| IEAAAG14 | (AAAG)_18_ | 195-203 | 208 |
| IEAAAG15 | (AAAG)_7_ | 108-128 | 119 |

^1^Expected product size from Bourke and Dawson 2006

Table S10: Genomic composition of avian mitochondrial DNA

| Species | Family | Genbank | alpha |  | protein |  | rRNA |  | control |  |
| --- | --- | --- | --- | --- | --- | --- | --- | --- | --- | --- |
|  |  |  | Length | A+T | Length | A+T | Length | A+T | Length | A+T |
| *Aquila chrysaetos* | Accipitridae |  | 17,332 | 53 | 11,445 | 52 | 2,505 | 52 | 1,157 | 56 |
| *Spizaetus nipalensis* | Accipitridae | AP008238 | 17,667 | 53 | 11,337 | 52 | 2,514 | 52 | 1,158 | 57 |
| *Accipiter gentilis* | Accipitridae | AP010797 | 18,266 | 56 | 11,346 | 54 | 2,557 | 55 | 1,977 | 62 |
| *Falco peregrinus* | Falconidae | NC_000878 | 18,068 | 56 | 11,367 | 54 | 2,578 | 53 | 1,510 | 57 |
| *Gallus gallus* | Phasianidae | AP003580 | 16,788 | 54 | 11,393 | 53 | 2,601 | 53 | 1,232 | 60 |
